# Key molecules associated with thyroid carcinoma prognosis: A study based on transcriptome sequencing and GEO datasets

**DOI:** 10.3389/fimmu.2022.964891

**Published:** 2022-08-17

**Authors:** Miaoyu Bai, Shanjia Ke, Hongjun Yu, Yanan Xu, Yue Yu, Shounan Lu, Chaoqun Wang, Jingjing Huang, Yong Ma, Wenjie Dai, Yaohua Wu

**Affiliations:** ^1^ Department of Minimal Invasive Hepatic Surgery, The First Affiliated Hospital of Harbin Medical University, Harbin, China; ^2^ Key Laboratory of Hepatosplenic Surgery, Ministry of Education, The First Affiliated Hospital of Harbin Medical University, Harbin, China; ^3^ Department of Hepatopancreatobiliary Surgery, Affiliated Hangzhou First People’s Hospital, Zhejiang University School of Medicine, Hangzhou, China; ^4^ Department of Thyroid Surgery, The First Affiliated Hospital of Harbin Medical University, Harbin, China

**Keywords:** RGS8, DGKI, OCA2, thyroid carcinoma, prognosis, immune infiltration, DNA methylation, transcriptome sequencing

## Abstract

**Background:**

Thyroid carcinoma (THCA) has a low mortality rate, but its incidence has been rising over the years. We need to pay attention to its progression and prognosis. In this study, a transcriptome sequencing analysis and bioinformatics methods were used to screen key genes associated with THCA development and analyse their clinical significance and diagnostic value.

**Methods:**

We collected 10 pairs of THCA tissues and noncancerous tissues, these samples were used for transcriptome sequencing to identify disordered genes. The gene expression profiles were obtained from the Gene Expression Omnibus (GEO) database. Comprehensive analysis of thyroid clinicopathological data using The Cancer Genome Atlas (TCGA). R software was used to carry out background correction, normalization and log2 conversion. We used quantitative real-time PCR (qRT–PCR) and Western blot to determine differentially expressed genes (DEGs) expression in samples. We integrated the DEGs expression, clinical features and progression-free interval (PFI). The related functions and immune infiltration degree were established by Gene Ontology (GO), Kyoto Encyclopedia of Genes and Genomes (KEGG), Gene Set Enrichment Analysis (GSEA), and single-sample Gene Set Enrichment Analysis (ssGSEA). The UALCAN database was used to analyse the methylation level.

**Results:**

We evaluated DEGs between normal tissue and cancer. Three genes were identified: regulator of G protein signaling 8 (RGS8), diacylglycerol kinase iota (DGKI) and oculocutaneous albinism II (OCA2). The mRNA and protein expression levels of RGS8, DGKI and OCA2 in normal tissues were higher than those in THCA tissues. Better survival outcomes were associated with higher expression of RGS8 (HR=0.38, P=0.001), DGKI (HR=0.52, P=0.022), and OCA2 (HR=0.41, P=0.003). The GO analysis, KEGG analysis and GSEA proved that the coexpressed genes of RGS8, DGKI and OCA2 were related to thyroid hormone production and peripheral downstream signal transduction effects. The expression levels of RGS8, DGKI and OCA2 were linked to the infiltration of immune cells such as DC cells. The DNA methylation level of OCA2 in cancer tissues was higher than that in the normal samples.

**Conclusions:**

RGS8, DGKI and OCA2 might be promising prognostic molecular markers in patients with THCA and reveal the clinical significance of RGS8, DGKI and OCA2 in THCA.

## Introduction

Over the past four decades, thyroid cancer morbidity has continued to increase worldwide (1). Thyroid cancer is the most common endocrine malignancy and accounts for 3.4% of all cancers diagnosed each year (2). More than 90% of thyroid cancers are papillary thyroid carcinoma (PTC) and follicular thyroid carcinoma. Medullary thyroid carcinoma and poorly differentiated thyroid cancer account for 5% each, and the proportion of anaplastic thyroid cancer (1%) is the lowest. Most patients with PTC have a good prognosis in terms of long-term survival (3), but the overall survival of patients with aggressive PTC (such as lymph node and distant metastasis potential) remains unsatisfactory (4). Therefore, revealing the molecular mechanism underlying PTC development may provide a new therapeutic direction for PTC treatment.

As tumor biology advances, genetic markers can be quantified through standardized tests to dynamically reflect the prognosis of patients based on their disease. Importantly, these genes may also play a crucial role in the progression of PTC. Furthermore, such genes may be potential targets for inhibiting recurrence and metastasis.

This study performed a bioinformatics analysis of the DEGs based on transcriptome sequencing and biological information from GEO datasets. Here, our aim is to lay a solid foundation for exploring the molecular mechanisms of THCA pathogenesis and identifying molecular targets for clinical diagnosis or treatment. In this study, we demonstrated the clinical correlation, potential diagnostic and prognostic effect of RGS8, DGKI and OCA2 in THCA using statistical and bioinformatics methods.

## Materials and methods

### Transcriptome sequencing

From January 2020 to December 2021, we collected 10 pairs of papillary thyroid carcinoma and adjacent thyroid tissues from patients who underwent thyroid resection at the First Affiliated Hospital of Harbin Medical University (**Supplementary Table 1**). Before surgery, these patients were not treated with chemotherapy or radiotherapy. With the approval of the Ethics Committee of the First Affiliated Hospital of Harbin Medical University, we collected specimens from patients after obtaining their informed consent. Then, we used these 10 samples for transcriptome sequencing to identify genes that were dysregulated.

### GEO and TCGA cohorts

The GSE33630 (45 normal thyroid tissues and 49 papillary thyroid cancer tissues) and GSE29265 (20 normal thyroid tissues and 20 papillary thyroid cancer tissues) gene expression profile matrix files were downloaded from the GEO database (https://www.ncbi.nlm.nih.gov/geo) (5). The Impute and Limma packages (version: 3.42.2) in R software (version: 3.6.3) were applied to perform background correction, normalization and log2 conversion of the matrix data of our sequencing results and each GEO dataset. We defined a |log2-fold change|≥2 and adjusted p value<0.05 as the DEGs screening criteria for the thyroid carcinoma samples from our sequencing results and two GEO datasets and then generated heatmaps (R package: ComplexHeatmap), volcano maps (R package: ggplot2) and venn diagrams (R package: ggplot2). Using TCGA (https://portal.gdc.cancer.gov) (6), standardized RNA-seq data (TPM) and corresponding clinical characteristics of PTC patients were downloaded.

### Clinical analysis and assessment of the prognostic status

We studied the clinical pathological factors associated with the 10 years PFI in TCGA specimens using the Kaplan–Meier method and Cox regression by R (package: survminer) (7). R (package: pROC) was used to analyse and visualize the receiver operating characteristic curve (ROC) of the genes involved in thyroid cancer, analyse their molecular expression and predict the predictive efficacy of the outcome.

### Correlation and enrichment analyses

We used the EnrichGO function (R package: clusterProfiler) to perform a GO analysis and then used the EnrichKEGG function (R package: clusterProfiler) to perform a KEGG analysis. Similarly, a GSEA was performed using the gseGO, gseKEGG, and gsePathway functions (R package: clusterProfiler) (8), detailed descriptions of individual gene sets are available in MSigDB Collections. We analysed the correlation between RGS8, DGKI and OCA2 and other related genes in thyroid cancer using TCGA data. The related genes were selected for an enrichment analysis.

### Immune cell infiltration

The immune invasiveness of RGS8, DGKI and OCA2 across cancers was analysed using TISIDB (http://cis.hku.hk/TISIDB) (9). The proportional infiltration levels of 24 types of immune cells were quantified using an ssGSEA, and Spearman correlation and Wilcoxon tests were used to evaluate the correlation between RGS8, DGKI and OCA2 expression and immune cell infiltration in different groups (10, 11).

### Methylation analysis

The UALCAN online database (http://ualcan.path.uab.edu) is a web portal used to perform survival analyses based on DNA methylation biomarkers supported by TCGA data (12). UALCAN was used to analyse the methylation levels of OCA2. The statistical analysis and visualization of the CpG site of OCA2 were performed using the R package (V 3.6.3).

### Western blot

The experimental method has been described before (13). The extracted tissues were denatured by electrophoresis and then transferred to a polyvinylidene fluoride membrane (Merck Millipore Ltd., Germany). The membrane was blocked with 5% skim milk and incubated with primary antibodies at 4°C overnight. Finally, the membrane was incubated with IRDye 800CW secondary antibodies (LI-COR, USA) (1:10000) at room temperature for 1 h, and the proteins were visualized and analyzed with Odyssey^®^ Imaging System (LI-COR, USA). Primary antibodies against the following target proteins were used: RGS8 (Santa) (1:500), DGKI (Santa) (1:500), OCA2 (Novus) (1:500) and GAPDH (Sigma-Al-drich) (1:8000).

### Quantitative real-time PCR

We used an AxyPrep Multisource Total RNA Miniprep Kit (Axygen Scientific, Inc, USA) to extract the total RNA from tissues and then reverse transcribed the RNA into cDNA using a PCR RT Kit (TOYOBO, Shanghai, China) after the RNA quantification. Quantitative real-time PCR was performed on an ABIPRISM 7500HT instrument (Applied Biosystems) using THUNDERBIRD SYBR qPCR Mix (TOYOBO, Shanghai, China) according to the manufacturer’s instructions to detect the expression of mRNA. The following primers were used: RGS8-F, 5’-GGATGCCTGTCTCACAAGTCA-3’; RGS8-R, 5’-CCTCGTAGCTTCTTCTGTCGATA-3’; DGKI-F, 5’-GCAGGTCTCGTACAGGAAAGC-3’; DGKI-R, 5’-CACTCCAGTCCAAAGTCGCTC-3’; OCA2-F, 5’-GCGGACTCGCTGAACTTGT-3’; OCA2-R, 5’-AGAAGAGTGAGACCTCCCTTTT-3’; GAPDH-F, 5’-TGACTTCAACAGCGACACCCA-3’; GAPDH-R, 5’-CACCCTGTTGCTGTAGCCAAA-3’. GAPDH was used as a control to determine changes in mRNA levels using the -ΔΔCT method.

## Results

### Identification of DEGs

**Figure 1:**
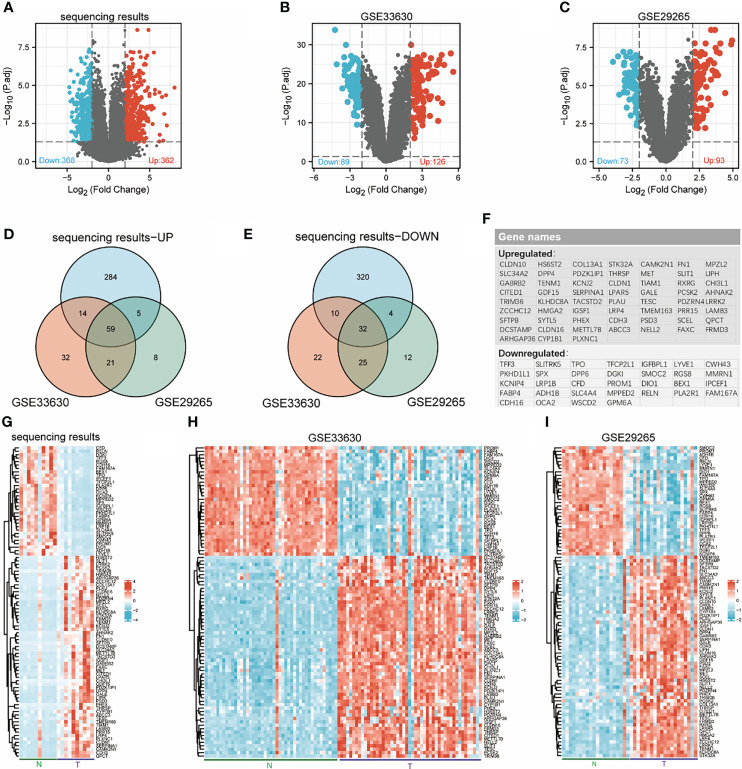
Identification of differentially expressed genes (DEGs). Volcanic maps for each data set: downregulated genes (blue dots) and upregulated genes (red dots) in THCA compared with normal thyroid; transcriptome sequencing results **(A)**, GSE33630 **(B)**, GSE29265 **(C)**. Venn diagrams: 59 overlapping upregulated genes in sequencing data, GSE33630 and GSE29265 **(D)**, 32 overlapping downregulated genes in sequencing data, GSE33630 and GSE29265 **(E)**. The names of 91 overlapping differentially expressed genes **(F)**. Heatmaps for each data set show the expression data of 91 genes: transcriptome sequencing results **(G)**, GSE33630 **(H)**, GSE29265 **(I)**.

Ten pairs of thyroid carcinoma specimens were used for transcriptome sequencing, the edgeR package was utilized to assess the DEGs between normal tissue and cancer with the cut-off values of |log2-fold change|≥2 and adjusted p value< 0.05. In total, 730 genes (362 upregulated and 368 downregulated) were identified between THCA and normal samples (**Figure 1A**). Then, we searched and obtained two gene expression profiles from GSE33630 and GSE29265, further validating the 730 genes. The two GEO datasets, GSE33630 and GSE29265, included in this study are shown in **Figures 1B, C**. According to the Venn diagrams and heatmaps (**Figures 1D, E, G–I**), we found 91 differentially expressed genes. We screened 32 genes in the three cogroups found to be repeatedly downregulated and 59 genes found to be repeatedly upregulated in THCA from the three cohorts (**Figure 1F**). Furthermore, we performed biological function correlation analysis using the TCGA data, and the results showed that the downregulated genes were highly correlated with thyroid function as shown in **Supplementary Figures 1A, B**.

### Expression of RGS8, DGKI and OCA2 was decreased in THCA tissues

**Figure 2:**
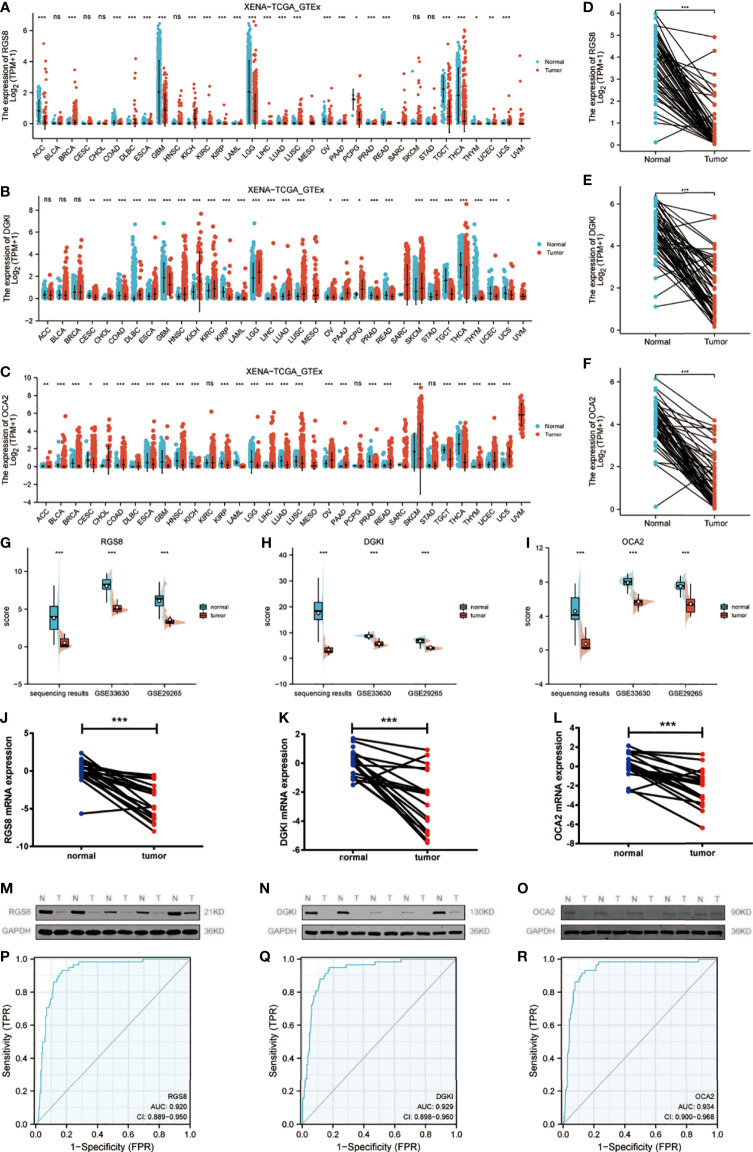
Expression of RGS8, DGKI and OCA2 was decreased in THCA tissues. In different cancers in TCGA and GTEx databases, the upregulation and downregulation levels of RGS8 **(A)**, DGKI **(B)**, OCA2 **(C)**. The expression levels of RGS8 **(D)**, DGKI **(E)**, OCA2 **(F)** in THCA, n=58. The expression levels of RGS8 **(G)**, DGKI **(H)**, OCA2 **(I)** in transcriptome sequencing results, GSE33630 and GSE29265. The expression levels of RGS8 **(J)**, DGKI **(K)**, OCA2 **(L)** in 20 pairs of papillary thyroid carcinoma tissues and adjacent normal tissues were detected by qRT–PCR. The expression levels of RGS8 **(M)**, DGKI **(N)**, OCA2 **(O)** in 5 pairs of papillary thyroid carcinoma tissues and adjacent normal tissues were detected by western blotting. The ROC curve of RGS8 **(P)**, DGKI **(Q)**, OCA2 **(R)**. *p < 0.05, **p < 0.01, ***p < 0.001, NS, no significance.

We used qRT–PCR to detect some unreported genes among 91 genes in tumor tissues and adjacent noncancer tissues from 20 patients with papillary thyroid carcinoma. The results showed that the expression levels of RGS8, DGKI and OCA2 were obviously reduced in the THCA tissues compared with the corresponding noncancerous tissues (**Figures 2J–L**). Then, we detected the protein expression of RGS8, DGKI and OCA2 in five pairs of papillary thyroid carcinoma tissues and found that their expression levels were obviously lower than those of the paired normal thyroid tissues (**Figures 2M–O**). On the basis of TCGA and Genotype-Tissue Expression (GTEx) datasets, we used a Wilcoxon test to analyse the data. We first evaluated RGS8, DGKI, and OCA2 expression across cancers. The expression of RGS8, DGKI, and OCA2 was decreased in most tumors, including THCA (**Figures 2A–C**). Similarly, by analysing RGS8, DGKI, and OCA2 expression in the TCGA database, we found that their expression in 58 THCA tumor samples was lower than that in 58 paired normal samples. (**Figures 2D–F**). In addition, RGS8, DGKI, and OCA2 were expressed at low levels in thyroid cancer in our sequencing results, GSE33630 and GSE29265 (**Figures 2G–I**). Additionally, we established a receiver operating characteristic (ROC) curve, and the areas under the curve (AUCs) of RGS8, DGKI and OCA2 were 0.920, 0.929 and 0.934 (**Figures 2P–R**), respectively, indicating a high diagnostic value.

### Correlation between RGS8, DGKI and OCA2 expression and clinical features

**Figure 3:**
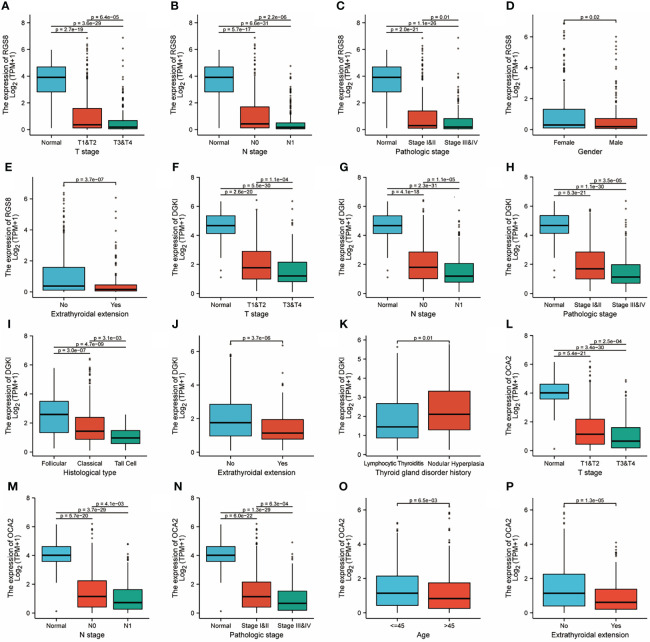
Clinicopathological features and expression of RGS8, DGKI and OCA2 in THCA. RGS8 expression level with T stage **(A)**, N stage **(B)**, pathological stage **(C)**, gender **(D)**, extrathyroidal extension **(E)**. DGKI expression level with T stage **(F)**, N stage **(G)**, pathological stage **(H)**, histological type **(I)**, extrathyroidal extension **(J)**, thyroid gland disorder history **(K)**. OCA2 expression level with T stage **(L)**, N stage **(M)**, pathological stage **(N)**, age **(O)**, extrathyroidal extension **(P)**.

To investigate the role of RGS8, DGKI and OCA2 expression, we explored the relationship between RGS8, DGKI, and OCA2 expression and clinical features in thyroid cancer patients, and the expression data were obtained from TCGA. As demonstrated in **Figures 3A–E**, the expression of RGS8 was associated with the T stage (T1 and 2 vs. T3 and 4, P=6.4e-05), N stage (N0 vs. N1, P=2.2e-06), pathological stage (stages III and IV vs. stage I and II, P=0.01), gender (female vs. male, P=0.02), and extrathyroidal extension (No vs. Yes, P=3.7e-07). As shown in **Figures 3F–K**, the expression level of DGKI was significantly related to the T stage (T1 and 2 vs. T3 and 4, P=1.1e-04), N stage (N0 vs. N1, P=1.1e-05), pathological stage (stages III and IV vs. stage I and II, P=3.5e-05), histological type (follicular vs. classical, P=3.0e-07, follicular vs. tall cell P=4.7e-09, classical vs. tall cell P=3.1e-03), extrathyroidal extension (No vs. Yes, P=3.7e-06), and thyroid gland disorder history (lymphocytic thyroiditis vs. nodular hyperplasia, P=0.01). The expression of OCA2 was correlated with the T stage (T1 and 2 vs. T3 and 4, P=2.5e-04), N stage (N0 vs. N1, P=4.1e-03), pathological stage (stages III and IV vs. stage I and II, P=6.3e-04), age (≤45 years vs. >45 years, P=6.5e-03), and extrathyroidal extension (No vs. Yes, P=1.3e-05) as shown in **Figures 3L–P**. The correlation between the expression of RGS8, DGKI and OCA2 and other clinicopathological features was shown in **Supplementary Tables 2**, **3** and **4**. Our research demonstrates that THCA patients with a low expression of RGS8, DGKI, and OCA2 were more likely to develop to a more advanced stage.

### High expression of RGS8, DGKI and OCA2 was significantly related to a better prognosis in patients with THCA

**Figure 4:**
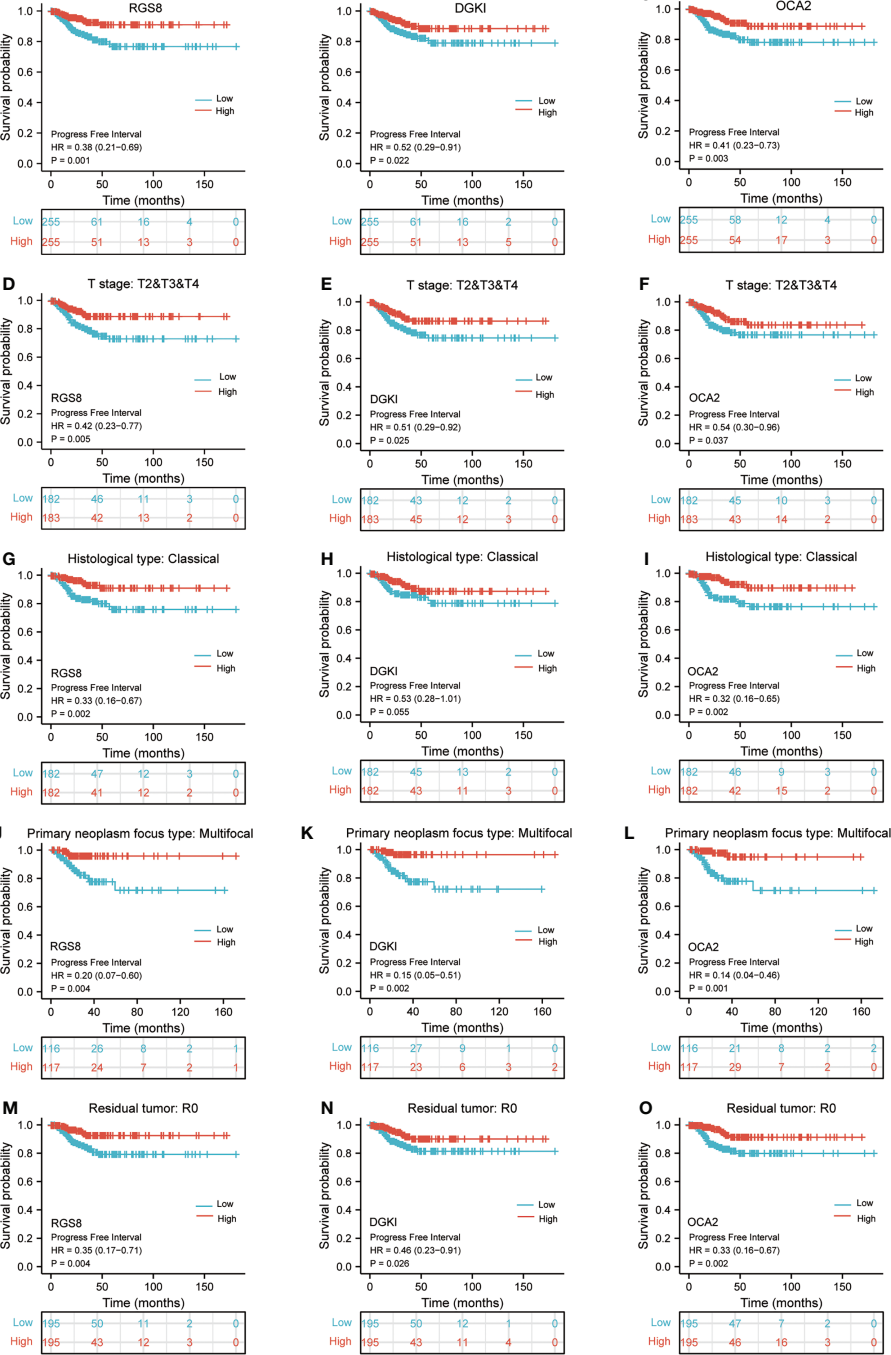
Kaplan–Meier survival plots comparing the low and high expression of RGS8, DGKI and OCA2 in THCA. PFI survival curves of patients with THCA between high and low expression of RGS8 **(A)**, DGKI **(B)** and OCA2 **(C)**. PFI survival curves of T stages 2, 3 and 4 between RGS8-high and -low patients **(D)**, DGKI-high and -low patients **(E)**, OCA2-high and -low patients **(F)** with THCA. PFI survival curves of classical histological type between RGS8-high and -low patients **(G)**, DGKI-high and -low patients **(H)**, OCA2-high and -low patients **(I)** with THCA. PFI survival curves of multifocal primary neoplasm focus type between RGS8-high and -low patients **(J)**, DGKI-high and -low patients **(K)**, OCA2-high and -low patients **(L)** with THCA. PFI survival curves of R0 between RGS8-high and -low patients **(M)**, DGKI-high and -low patients **(N)**, OCA2-high and -low patients **(O)** with THCA.

To determine whether the expression of RGS8, DGKI and OCA2 affect the prognosis of patients, we classified the THCA patients in the TCGA database and conducted a prognostic analysis on the basis of the expression values of RGS8, DGKI and OCA2. Among the THCA patients, the PFI rates in the RGS8 (HR=0.38, P=0.001), DGKI (HR=0.52, P=0.022), and OCA2 (HR=0.41, P=0.003) high expression group were obviously higher than those in the low expression group (**Figures 4A–C**). Then, based on the PFI, we performed a prognostic subgroup analysis, and the results showed that the PFI rates of the THCA samples with higher RGS8 (HR=0.42, P=0.005), DGKI (HR=0.51, P=0.025) and OCA2 (HR=0.54, P=0.037) expression were better in the T stage (T 2/3/4), the PFI rates of the THCA samples with higher RGS8 (HR=0.33, P=0.002), DGKI (HR=0.53, P=0.055) and OCA2 (HR=0.32, P=0.002) were better in the histological type (classical), the PFI rates of the THCA samples with higher RGS8 (HR=0.20, P=0.004), DGKI (HR=0.15, P=0.002) and OCA2 (HR=0.14, P=0.001) expression were better in the primary neoplasm focus type (multifocal) and that the PFI rates of the THCA samples with higher RGS8 (HR=0.35, P=0.004), DGKI (HR=0.46, P=0.026) and OCA2 (HR=0.33, P=0.002) expression were better in the residual tumor (R0) (**Figures 4D–O**). In univariate analysis, RGS8, DGKI and OCA2 expression were associated with PFI (**Supplementary Table 5**). These data demonstrate that an increased expression of RGS8, DGKI, and OCA2 is associated with a better PFI, and it has the ability to predict prognosis in some specific types of thyroid cancer.

### Enrichment of biofunction and associated genes analysis of RGS8, DGKI and OCA2 in THCA

**Figure 5:**
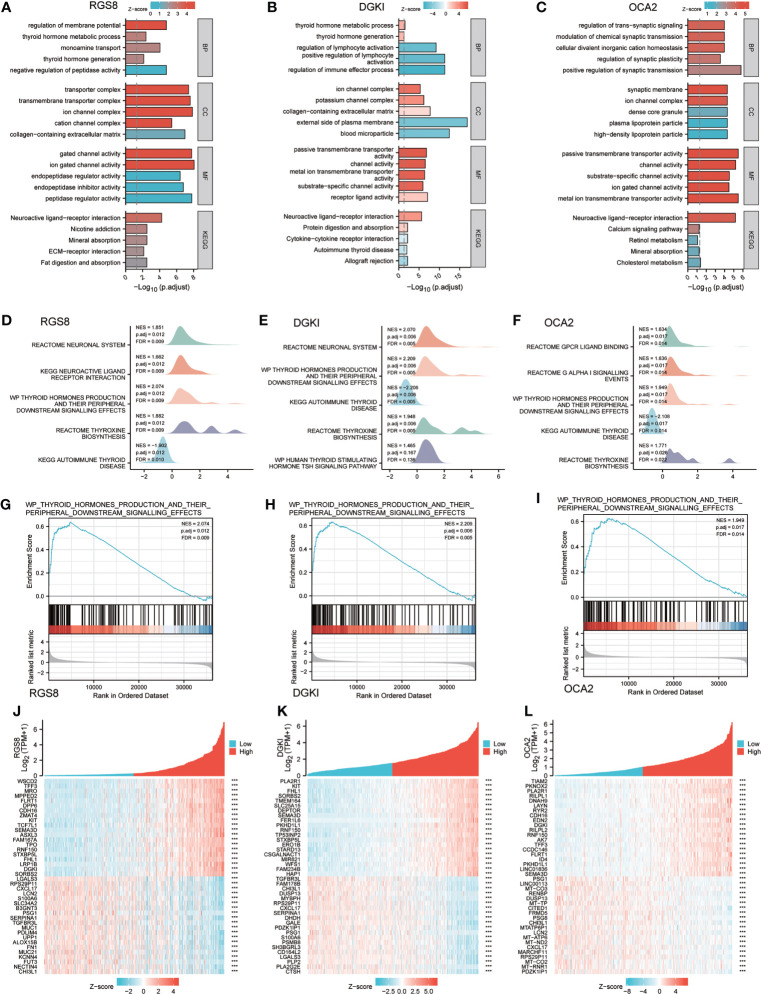
Enrichment of biofunction and associated genes analysis of RGS8, DGKI and OCA2 in THCA. The GOKEGG analysis of RGS8 **(A)**, DGKI **(B)**, OCA2 **(C)**. The GSEA of RGS8 **(D)**, DGKI **(E)**, OCA2 **(F)**, and the thyroid hormones production and their peripheral downstream signalling effects of RGS8 **(G)**, DGKI **(H)**, OCA2 **(I)** in GSEA analysis. Heatmap of coexpression patterns of RGS8 **(J)**, DGKI **(K)** and OCA2 **(L)** related genes. ***p < 0.001.

To analyse the biological function and associated pathways of RGS8-related genes, DGKI-related genes and OCA2-related genes, we performed a biological function correlation analysis using TCGA data, and the results demonstrated that RGS8-, DGKI- and OCA2- related genes were involved in numerous of biological processes (BPs), cellular compositions (CCs), molecular functions (MFs) and KEGG pathways. Regarding the GO analysis, RGS8 was mostly enriched in the regulation of the membrane potential, transporter complex, ion gated channel activity and more importantly, thyroid hormone generation and thyroid hormone metabolic process (**Figure 5A**). The differential expression of DGKI could modulate the regulation of the immune effector process, the external side of the plasma membrane, and passive transmembrane transporter activity, including thyroid hormone generation and thyroid hormone metabolic process (**Figure 5B**). OCA2 is mainly related to cellular divalent inorganic cation homeostasis, synaptic membrane, passive transmembrane transporter activity and neuroactive ligand–receptor interaction (**Figure 5C**).

To explore RGS8-, DGKI- and OCA2- associated pathways in THCA, we performed a GSEA using gene sets from the Molecular Signatures Database Collection (MSigDB) (c2.cp.reactome/biocarta/kegg.v6.2.symbols.gmt). The enrichment plots of the GSEA showed that thyroid hormone production and its peripheral downstream signalling effects, thyroxine biosynthesis, and autoimmune thyroid disease were significantly enriched in RGS8, DGKI and OCA2 (**Figures 5D–I**). We also studied the coexpression pattern of RGS8, DGKI and OCA2 in THCA in the TCGA database. (**Figures 5J–L**).

### Correlation between RGS8, DGKI, OCA2 expression and immune characteristics

**Figure 6:**
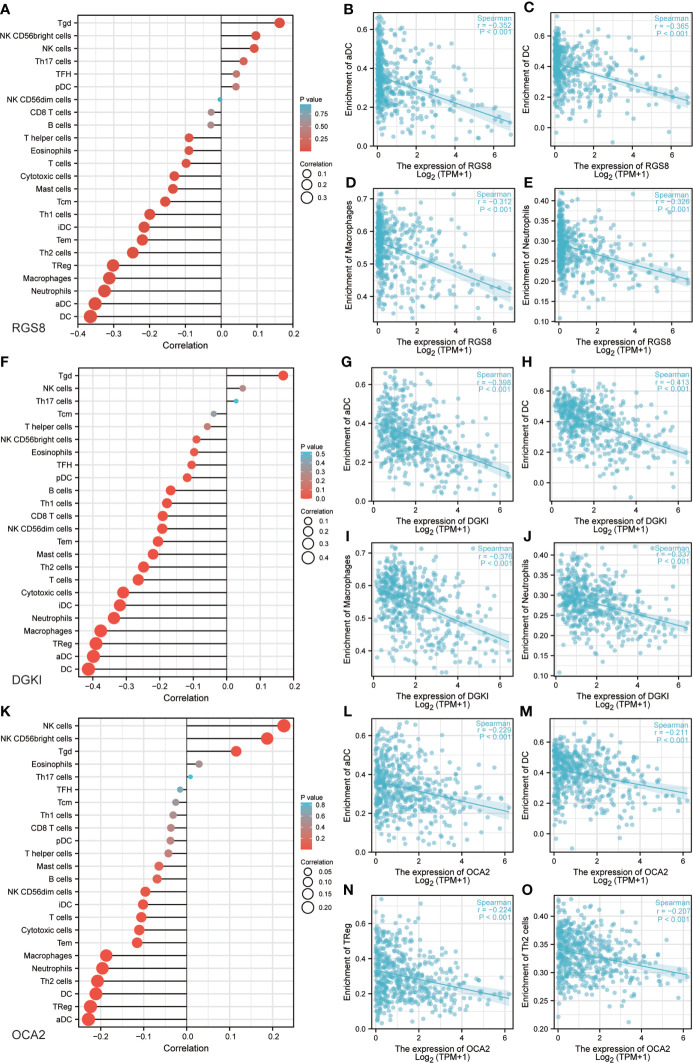
Correlation between RGS8, DGKI, OCA2 expression and immune characteristics. Association between the RGS8 **(A)**, DGKI **(F)**, OCA2 **(K)** expression level and relative abundances of 24 immune cells. Scatter plot of RGS8 **(B–E)**, DGKI **(G–J)**, OCA2 **(L–O)** expression and individual immune cell infiltration levels.

Previous studies have shown that tumor infiltrating immune cells (TIICs) play a crucial regulatory role in the occurrence and development of tumors. By quantifying ssGSEA in the THCA tumor environment, we applied the Spearman correlation to prove the correlation between the level of immune cell infiltration and the expression of RGS8, DGKI and OCA2. First, using TISIDB, we analysed the relationship between the expression levels of RGS8, DGKI and OCA2 and the level of immune cell infiltration in different cancers (**Supplementary Figures 1C–E**). As shown in **Figures 6A–E**, RGS8 expression was negatively correlated with activated DCs (R=-0.352, P<0.01), DCs (R=-0.365, P<0.01), macrophages (R=-0.312, P<0.01), and neutrophils (R=-0.326, P<0.01) and positively associated with T cells (R=0.163, P<0.01). Similarly, as shown in **Figures 6F–J**, DGKI expression was negatively correlated with activated DCs (R=-0.398, P<0.01), DCs (R=-0.413, P<0.01), macrophages (R=-0.376, P<0.01), neutrophils (R=-0.337, P<0.01) and positively associated with T cells (R=0.169, P<0.01). As illustrated in **Figures 6K–O**, OCA2 expression was negatively correlated with activated DCs (R=-0.229, P<0.01), DCs (R=-0.211, P<0.01), TRegs (R=-0.224, P<0.01), and Th2 cells (R=-0.207, P<0.01) and positively associated with NK cells (R=0.226, P<0.01).

### DNA methylation of OCA2

**Figure 7:**
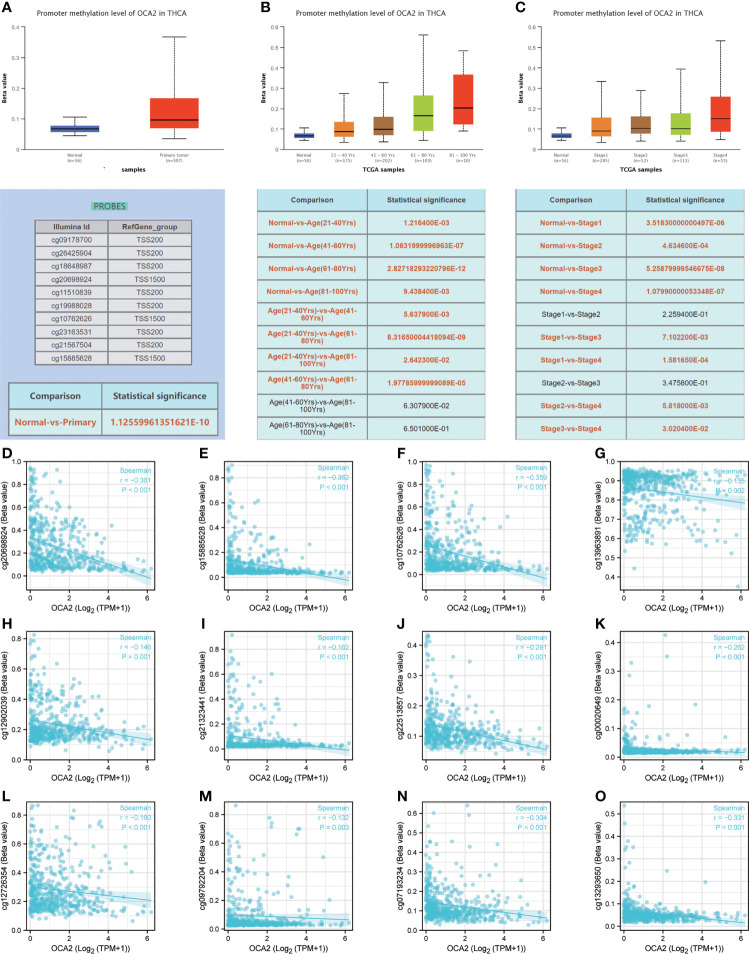
DNA methylation of OCA2. Expression of OCA2 DNA methylation level in THCA and normal tissues **(A)**. Expression of OCA2 DNA methylation levels in THCA patients of different ages **(B)**. Expression of OCA2 DNA methylation levels THCA patients with different pathologic stages **(C)**. Different CpG sites of OCA2 methylation levels in THCA **(D–O)**.

DNA methylation is an indispensable component of epigenetics and can silence the expression of methylated genes. To explore the cause of the low expression of OCA2 in THCA, the UALCAN online database was used to analyse the methylation status of OCA2. The DNA methylation level of OCA2 was expressively elevated in the THCA tumor tissue compared with that in normal tissue (**Figure 7A**). OCA2 is highly methylated in old age and advanced tumors (**Figures 7B, C**). Some predicted CpG sites of OCA2 were negatively correlated with the methylation levels (**Figures 7D–O**). These results suggest that abnormal methylation is a main cause of OCA2 gene silencing.

## Discussion

Thyroid carcinoma is the most common endocrine malignant tumor, among these; papillary thyroid carcinoma (PTC) is particularly important (14). On a global scale, the incidence of PTC has been rising (15). Although the prognosis of PTC is optimistic, the survival rate of PTC patients with invasive characteristics is not satisfactory (16). Currently, reliable and specific markers for the detection and staging of the disease are lacking, requiring additional surgery to obtain a definitive diagnosis (17). As a result, patients with PTC have the additional risks of surgical trauma and surgical complications. Traditional clinicopathological parameters, such as TNM staging, can forecast PTC-related mortality, but it is difficult to estimate the risk of recurrence (18). It is very urgent to explore the molecular mechanism underlying PTC and predict the prognosis of PTC.

In this study, 91 reliable differentially expressed genes were identified in PTC by sequencing and a comprehensive analysis of GEO and TCGA datasets. We experimentally verified these genes and found that the expression of RGS8, DGKI and OCA2 was closely related to THCA PFI.

G protein signal regulatory proteins were originally described as inhibitors of G protein-coupled receptor (GPCR)-induced signal transduction cascades and can enhance the inherent GTPase activity of heterotrimer G proteins (19). RGS8 expression seems to be mainly distributed in the brain (20) and is a brain-enriched protein. RGS8 has been reported in human breast cancer and pancreatic cancer (21, 22), and was downregulated in ovarian cancer (23), but the molecular mechanism remains unclear. RGS8 has not been reported to be associated with thyroid cancer.

Diacylglycerol (DAG) plays a central role in complex lipid synthesis and intracellular signal transduction. Diacylglycerol kinases are special lipid kinases that convert glycerol diacylglycerol to phosphatidic acid and terminate DAG-based signal transduction (24). Diacylglycerol kinase iota (DGKI) is a member of the subfamily of type IV diacylglycerol kinases and has been reported to be isolated from human retinas and brain libraries (25). The latest studies have reported that DGKI is overexpressed in colon, gastric and liver carcinomas and is linked to an unsatisfactory prognosis (26–28). DGKI has also been reported to be associated with a better prognosis in glioblastoma (29). Nevertheless, the association between DGKI and thyroid cancer has not been reported.

OCA2, also known as pink eye dilution protein or P-protein, is a protein with a crucial function in pigmentation (30). The OCA2 gene is homologous to the ion permeable membrane and encodes a pigment cell-specific 12 transmembrane domain protein (31). Mutations in the OCA2 gene have been proven to be related to an increased risk of skin melanoma or basal cell carcinoma (32, 33). OCA2 gene have been reported to be associated with overall survival among patients with estrogen receptor–negative tumors, with the rare G allele being associated with increased overall survival (34). However, the relationship between OCA2 and other carcinomas has not been reported.

In multiple databases, we found that these three genes are abnormally expressed in many tumors and significantly reduced in thyroid cancer. Then, we used qRT–PCR and a Western blot analysis to detect the expression of RGS8, DGKI and OCA2 in tumor tissues and corresponding noncancer tissues from THCA patients. The results showed that the mRNA and protein expression levels of RGS8, DGKI and OCA2 were obviously decreased in the THCA tissues compared to those in the adjacent noncancer tissues. The ROC scores of these three genes in the TCGA database were high, indicating a high diagnostic value.

To further investigate the significance and mechanism of RGS8, DGKI and OCA2 in thyroid cancer, we summarized the relationship between RGS8, DGKI and OCA2 expression and clinical features in the TCGA database of THCA patients. The results indicated that THCA patients with a low expression of RGS8, DGKI and OCA2 were more likely to develop advanced-stage. We also classified the THCA patients in the TCGA database and analysed their prognosis based on the expression values of RGS8, DGKI and OCA2. The PFI rate of the THCA patients with a high expression of RGS8, DGKI and OCA2 was obviously higher than that of the patients in the low expression group.

To explore the related pathways and biological functions of RGS8-, DGKI- and OCA2-related genes, a biological function correlation analysis was performed. The results showed that RGS8-, DGKI- and OCA2-related genes were involved in multiple BPs, CCs, MFs and KEGG pathways, including enrichment in thyroid hormone production and thyroid hormone metabolism. The GSEA showed that RGS8, DGKI and OCA2 were enriched in thyroid hormone production and its peripheral downstream signal transduction effects, thyroxine biosynthesis, and autoimmune thyroid diseases.

Research studies have reported that TIICs play an indispensable role in regulating the occurrence and development of tumors (35). The key to antitumor action is an effective immune response, but cancer cells have evolved various mechanisms that allow tumor cells to evade immune cell attacks, such as antigen presentation dysfunction and immunosuppressive cell collection (36). Previous studies have reported that immune infiltration can influence patient outcomes (37). Our results revealed that the expression of RGS8, DGKI, and OCA2 in thyroid carcinoma was negatively correlated with multiple types of immune cell infiltration, including dendritic cells (DCs and aDCs), macrophages and neutrophils. Many studies have proven that dendritic cells (38), macrophages (39) and neutrophils (40) play vital roles in the regulation of tumor development. These results explain why RGS8, DGKI and OCA2 may play important regulatory roles in the tumor immune microenvironment and THCA development.

In mammals, DNA methylation is a type of epigenetic modification that has been well studied. Methylation in normal cells can guarantee the proper regulation of gene expression and stable gene silencing (41). Proper DNA methylation is critical for development and normal cell function; thus, any abnormality in this process can lead to a variety of diseases, such as cancer. Numerous tumor suppressor genes silenced by DNA hypermethylation were found in tumor tissues. Undoubtedly, DNA methylation-related silencing can be a marker of cancer in humans because it plays a key role in tumors (42). We discovered that the DNA methylation level of OCA2 in cancer tissues was significantly higher than that in normal samples, suggesting that the high methylation level of OCA2 may be a reason for the low expression of OCA2 in THCA.

Although our study determined the prognostic value of RGS8, DGKI and OCA2 in THCA through a bioinformatics analysis, there are still some limitations and deficiencies. First, although we have identified the relationship between RGS8, DGKI and OCA2 expression levels and prognosis, their important regulatory mechanisms in THCA need to be further validated and evaluated by analyzing clinical samples from more centers, and the use of a single cohort to predict prognosis in public data sets is far from perfect. Second, in order to better assess the important role of genes in the progression of THCA, all types of clinical features should be involved, including treatment modalities in different patients, and loss of information in public databases was inevitable. The molecular mechanisms of RGS8, DGKI and OCA2 in THCA genesis and development have not been studied. Relevant experiments will be designed and implemented in our future research.

## Conclusions

In summary, this study proved that RGS8, DGKI and OCA2 are downregulated in THCA tissues and are closely related to the clinical stage, prognosis, immune infiltration and DNA methylation, which are valuable prognostic markers of THCA.

## Data availability statement

The datasets presented in this study can be found in online repositories. The names of the repository/repositories and accession number(s) can be found below: https://www.ncbi.nlm.nih.gov/-PRJNA861677

## Ethics statement

The studies involving human participants were reviewed and approved by the Ethics Committee of the First Affiliated Hospital of Harbin Medical University. The patients/participants provided their written informed consent to participate in this study.

## Author contributions

MB, SK, HY and YX made equal contributions to this article. MB, SK, HY planned the research trials and engaged in article writing. YX, CW, SL attended in information generation and analysis. JH, YY provided assistance with analysis. All authors contributed to the article and approved the submitted version.

## Funding

This work was supported by the Research Fund of the National Natural Scientific Foundation of China (81100305, 81470876, 81502605 and 81270527), Natural Science Foundation of Heilongjiang Province of China (QC2013C094, LC2018037), Chen Xiaoping Foundation for the Development of Science and Technology of Hubei Province (CXPJJH11900001-2019349), Outstanding Youth Training Fund from Academician Yu Weihan of Harbin Medical University (2014), and the First Affiliated Hospital of Harbin Medical University (2019L01, HYD2020JQ0007, HYD2020JQ0012).

## Acknowledgments

The authors thank the researchers, staff and patients who participated in the study.

## Conflict of interest

The authors declare that the research was conducted in the absence of any commercial or financial relationships that could be construed as a potential conflict of interest.

## Publisher’s note

All claims expressed in this article are solely those of the authors and do not necessarily represent those of their affiliated organizations, or those of the publisher, the editors and the reviewers. Any product that may be evaluated in this article, or claim that may be made by its manufacturer, is not guaranteed or endorsed by the publisher.

## Supplementary material

Supplementary Figure 1 The GOKEGG analysis of upregulated genes **(A)** and downregulated genes **(B)**. Relationship between the expression of RGS8 **(C)**, DGKI **(D)**, OCA2 **(E)** and the level of immune cell infiltration in different cancers.
