# Supplementary figures and images for: Key molecules associated with thyroid carcinoma prognosis: A study based on transcriptome sequencing and GEO datasets
Source: Front Immunol. 2022 Aug 17;13:964891. doi: 10.3389/fimmu.2022.964891 (PMC9428590; doi:10.3389/fimmu.2022.964891)

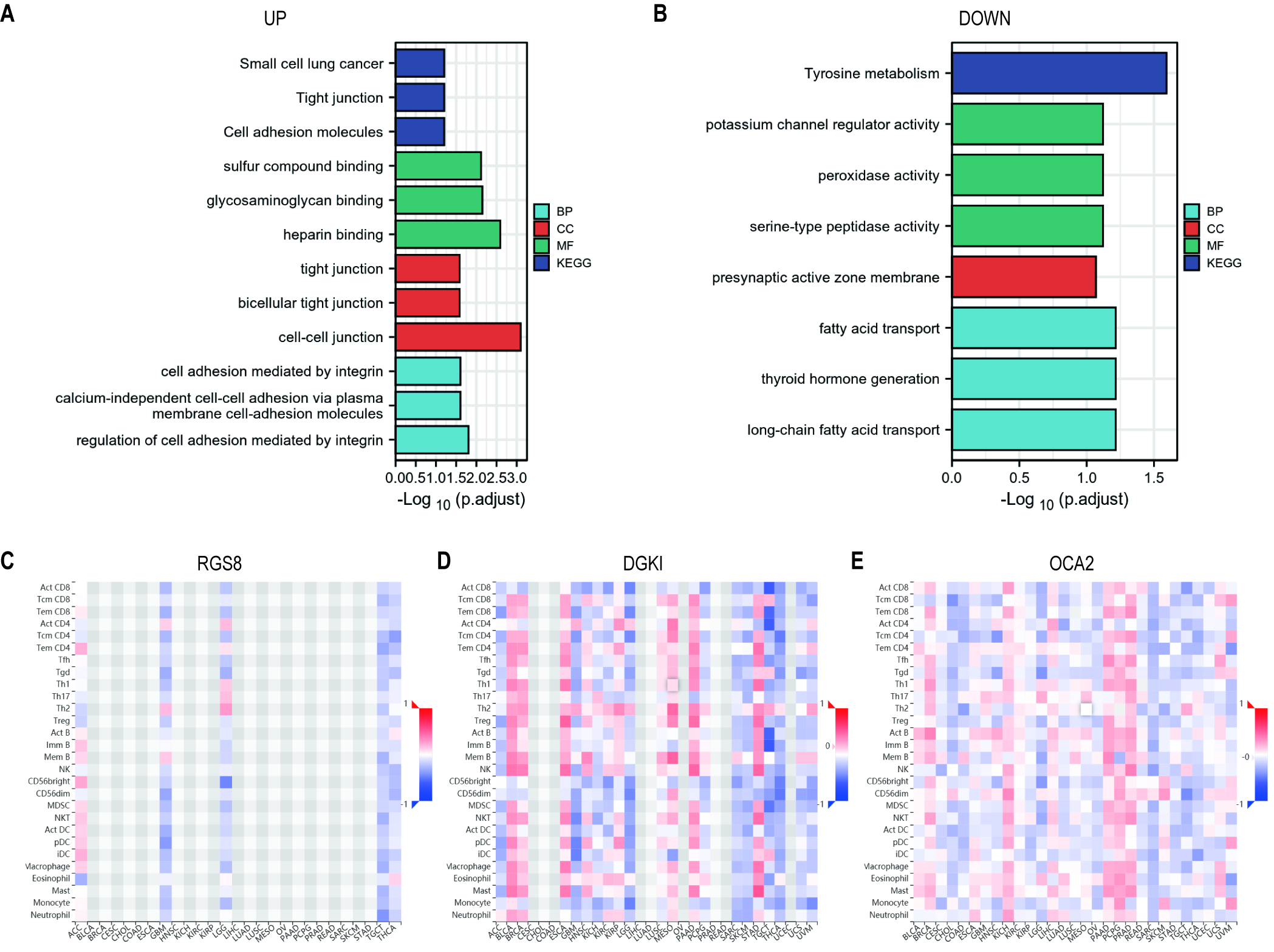

Supplement: Supplementary Figure 1 — The GOKEGG analysis of upregulated genes (A) and downregulated genes (B). Relationship between the expression of RGS8 (C), DGKI (D), OCA2 (E) and the level of immune cell infiltration in different cancers. [file Image_1.tif]
