# Supplementary material for: Key molecules associated with thyroid carcinoma prognosis: A study based on transcriptome sequencing and GEO datasets
Source: Front Immunol. 2022 Aug 17;13:964891. doi: 10.3389/fimmu.2022.964891 (PMC9428590; doi:10.3389/fimmu.2022.964891)
Supplement: Supplementary file 2 [file Table_1.docx]

| Number | Type | Age | Gender | Lymphatic metastasis | Distant metastasis | Recrudesce | Follow-up period |
| --- | --- | --- | --- | --- | --- | --- | --- |
| 1 | PTC | 47 | female | No | No | No | 2020-10-16 |
| 2 | PTC | 50 | female | No | No | No | 2020-10-22 |
| 3 | PTC | 44 | female | Yes | No | No | 2020-10-23 |
| 4 | PTC | 46 | male | Yes | No | No | 2020-10-28 |
| 5 | PTC | 27 | male | Yes | No | No | 2020-10-29 |
| 6 | PTC | 42 | female | Yes | No | No | 2020-12-29 |
| 7 | PTC | 16 | male | Yes | No | No | 2020-12-10 |
| 8 | PTC | 60 | male | Yes | No | No | 2020-12-15 |
| 9 | PTC | 38 | female | Yes | No | No | 2020-12-22 |
| 10 | PTC | 30 | female | Yes | No | No | 2020-10-15 |

**Supplementary Table 1.** 10 pairs of thyroid papillary carcinoma and adjacent thyroid tissues from patients undergoing thyroidectomy at the First Affiliated Hospital of Harbin Medical University from January 2020 to December 2021.
