# Supplementary material for: Key molecules associated with thyroid carcinoma prognosis: A study based on transcriptome sequencing and GEO datasets
Source: Front Immunol. 2022 Aug 17;13:964891. doi: 10.3389/fimmu.2022.964891 (PMC9428590; doi:10.3389/fimmu.2022.964891)
Supplement: Supplementary file 3 [file Table_2.docx]

| Characteristic | Low expression of RGS8 | High expression of RGS8 | p |
| --- | --- | --- | --- |
| n | 255 | 255 |  |
| T stage, n (%) |  |  | < 0.001 |
| T1 | 54 (10.6%) | 89 (17.5%) |  |
| T2 | 79 (15.6%) | 88 (17.3%) |  |
| T3 | 99 (19.5%) | 76 (15%) |  |
| T4 | 22 (4.3%) | 1 (0.2%) |  |
| N stage, n (%) |  |  | < 0.001 |
| N0 | 90 (19.6%) | 139 (30.2%) |  |
| N1 | 148 (32.2%) | 83 (18%) |  |
| M stage, n (%) |  |  | 0.502 |
| M0 | 145 (49.2%) | 141 (47.8%) |  |
| M1 | 6 (2%) | 3 (1%) |  |
| Pathologic stage, n (%) |  |  | 0.002 |
| Stage I | 136 (26.8%) | 150 (29.5%) |  |
| Stage II | 20 (3.9%) | 32 (6.3%) |  |
| Stage III | 57 (11.2%) | 56 (11%) |  |
| Stage IV | 41 (8.1%) | 16 (3.1%) |  |
| Gender, n (%) |  |  | 0.029 |
| Female | 174 (34.1%) | 197 (38.6%) |  |
| Male | 81 (15.9%) | 58 (11.4%) |  |
| Race, n (%) |  |  | 0.989 |
| Asian | 26 (6.3%) | 25 (6%) |  |
| Black or African American | 14 (3.4%) | 13 (3.1%) |  |
| White | 175 (42.3%) | 161 (38.9%) |  |
| Age, n (%) |  |  | 0.859 |
| <=45 | 122 (23.9%) | 119 (23.3%) |  |
| >45 | 133 (26.1%) | 136 (26.7%) |  |
| Histological type, n (%) |  |  | < 0.001 |
| Classical | 198 (38.8%) | 166 (32.5%) |  |
| Follicular | 28 (5.5%) | 73 (14.3%) |  |
| Other | 3 (0.6%) | 6 (1.2%) |  |
| Tall Cell | 26 (5.1%) | 10 (2%) |  |
| Residual tumor, n (%) |  |  | 0.043 |
| R0 | 190 (42.4%) | 200 (44.6%) |  |
| R1 | 32 (7.1%) | 22 (4.9%) |  |
| R2 | 4 (0.9%) | 0 (0%) |  |
| Extrathyroidal extension, n (%) |  |  | < 0.001 |
| No | 144 (29.3%) | 194 (39.4%) |  |
| Yes | 101 (20.5%) | 53 (10.8%) |  |
| Primary neoplasm focus type, n (%) |  |  | 0.246 |
| Multifocal | 110 (22%) | 123 (24.6%) |  |
| Unifocal | 141 (28.2%) | 126 (25.2%) |  |
| Neoplasm location, n (%) |  |  | 0.725 |
| Bilateral | 41 (8.1%) | 47 (9.3%) |  |
| Isthmus | 13 (2.6%) | 9 (1.8%) |  |
| Left lobe | 91 (18.1%) | 86 (17.1%) |  |
| Right lobe | 107 (21.2%) | 110 (21.8%) |  |
| Thyroid gland disorder history, n (%) |  |  | 0.167 |
| Lymphocytic Thyroiditis | 37 (8.2%) | 37 (8.2%) |  |
| Nodular Hyperplasia | 26 (5.8%) | 42 (9.3%) |  |
| Normal | 152 (33.6%) | 133 (29.4%) |  |
| Other, specify | 12 (2.7%) | 13 (2.9%) |  |
| OS event, n (%) |  |  | 1.000 |
| Alive | 247 (48.4%) | 247 (48.4%) |  |
| Dead | 8 (1.6%) | 8 (1.6%) |  |
| PFI event, n (%) |  |  | < 0.001 |
| Alive | 216 (42.4%) | 240 (47.1%) |  |
| Dead | 39 (7.6%) | 15 (2.9%) |  |
| Age, median (IQR) | 46 (34.5, 58) | 46 (36, 58) | 0.788 |

**Supplementary Table 2.** Association between RGS8 expression and clinicopathologic features in the validation cohort.
