# Supplementary material for: Key molecules associated with thyroid carcinoma prognosis: A study based on transcriptome sequencing and GEO datasets
Source: Front Immunol. 2022 Aug 17;13:964891. doi: 10.3389/fimmu.2022.964891 (PMC9428590; doi:10.3389/fimmu.2022.964891)
Supplement: Supplementary file 4 [file Table_3.docx]

| Characteristic | Low expression of DGKI | High expression of DGKI | p |
| --- | --- | --- | --- |
| n | 255 | 255 |  |
| T stage, n (%) |  |  | < 0.001 |
| T1 | 58 (11.4%) | 85 (16.7%) |  |
| T2 | 74 (14.6%) | 93 (18.3%) |  |
| T3 | 104 (20.5%) | 71 (14%) |  |
| T4 | 19 (3.7%) | 4 (0.8%) |  |
| N stage, n (%) |  |  | < 0.001 |
| N0 | 97 (21.1%) | 132 (28.7%) |  |
| N1 | 143 (31.1%) | 88 (19.1%) |  |
| M stage, n (%) |  |  | 1.000 |
| M0 | 153 (51.9%) | 133 (45.1%) |  |
| M1 | 5 (1.7%) | 4 (1.4%) |  |
| Pathologic stage, n (%) |  |  | 0.001 |
| Stage I | 132 (26%) | 154 (30.3%) |  |
| Stage II | 19 (3.7%) | 33 (6.5%) |  |
| Stage III | 65 (12.8%) | 48 (9.4%) |  |
| Stage IV | 39 (7.7%) | 18 (3.5%) |  |
| Gender, n (%) |  |  | 0.426 |
| Female | 181 (35.5%) | 190 (37.3%) |  |
| Male | 74 (14.5%) | 65 (12.7%) |  |
| Race, n (%) |  |  | 0.295 |
| Asian | 22 (5.3%) | 29 (7%) |  |
| Black or African American | 16 (3.9%) | 11 (2.7%) |  |
| White | 180 (43.5%) | 156 (37.7%) |  |
| Age, n (%) |  |  | 0.156 |
| <=45 | 112 (22%) | 129 (25.3%) |  |
| >45 | 143 (28%) | 126 (24.7%) |  |
| Histological type, n (%) |  |  | < 0.001 |
| Classical | 194 (38%) | 170 (33.3%) |  |
| Follicular | 28 (5.5%) | 73 (14.3%) |  |
| Other | 5 (1%) | 4 (0.8%) |  |
| Tall Cell | 28 (5.5%) | 8 (1.6%) |  |
| Residual tumor, n (%) |  |  | 0.145 |
| R0 | 191 (42.6%) | 199 (44.4%) |  |
| R1 | 28 (6.2%) | 26 (5.8%) |  |
| R2 | 4 (0.9%) | 0 (0%) |  |
| Extrathyroidal extension, n (%) |  |  | < 0.001 |
| No | 146 (29.7%) | 192 (39%) |  |
| Yes | 101 (20.5%) | 53 (10.8%) |  |
| Primary neoplasm focus type, n (%) |  |  | 0.246 |
| Multifocal | 110 (22%) | 123 (24.6%) |  |
| Unifocal | 141 (28.2%) | 126 (25.2%) |  |
| Neoplasm location, n (%) |  |  | 0.661 |
| Bilateral | 40 (7.9%) | 48 (9.5%) |  |
| Isthmus | 13 (2.6%) | 9 (1.8%) |  |
| Left lobe | 91 (18.1%) | 86 (17.1%) |  |
| Right lobe | 109 (21.6%) | 108 (21.4%) |  |
| Thyroid gland disorder history, n (%) |  |  | 0.010 |
| Lymphocytic Thyroiditis | 38 (8.4%) | 36 (8%) |  |
| Nodular Hyperplasia | 22 (4.9%) | 46 (10.2%) |  |
| Normal | 150 (33.2%) | 135 (29.9%) |  |
| Other, specify | 16 (3.5%) | 9 (2%) |  |
| OS event, n (%) |  |  | 0.446 |
| Alive | 249 (48.8%) | 245 (48%) |  |
| Dead | 6 (1.2%) | 10 (2%) |  |
| PFI event, n (%) |  |  | 0.014 |
| Alive | 219 (42.9%) | 237 (46.5%) |  |
| Dead | 36 (7.1%) | 18 (3.5%) |  |
| Age, median (IQR) | 48 (36, 60) | 45 (34, 57) | 0.168 |

**Supplementary Table 3.** Association between DGKI expression and clinicopathologic features in the validation cohort.
