# Supplementary material for: Key molecules associated with thyroid carcinoma prognosis: A study based on transcriptome sequencing and GEO datasets
Source: Front Immunol. 2022 Aug 17;13:964891. doi: 10.3389/fimmu.2022.964891 (PMC9428590; doi:10.3389/fimmu.2022.964891)
Supplement: Supplementary file 5 [file Table_4.docx]

| Characteristic | Low expression of OCA2 | High expression of OCA2 | p |
| --- | --- | --- | --- |
| n | 255 | 255 |  |
| T stage, n (%) |  |  | < 0.001 |
| T1 | 64 (12.6%) | 79 (15.6%) |  |
| T2 | 73 (14.4%) | 94 (18.5%) |  |
| T3 | 97 (19.1%) | 78 (15.4%) |  |
| T4 | 21 (4.1%) | 2 (0.4%) |  |
| N stage, n (%) |  |  | 0.009 |
| N0 | 103 (22.4%) | 126 (27.4%) |  |
| N1 | 133 (28.9%) | 98 (21.3%) |  |
| M stage, n (%) |  |  | 0.319 |
| M0 | 135 (45.8%) | 151 (51.2%) |  |
| M1 | 6 (2%) | 3 (1%) |  |
| Pathologic stage, n (%) |  |  | 0.008 |
| Stage I | 130 (25.6%) | 156 (30.7%) |  |
| Stage II | 25 (4.9%) | 27 (5.3%) |  |
| Stage III | 59 (11.6%) | 54 (10.6%) |  |
| Stage IV | 40 (7.9%) | 17 (3.3%) |  |
| Gender, n (%) |  |  | 0.551 |
| Female | 189 (37.1%) | 182 (35.7%) |  |
| Male | 66 (12.9%) | 73 (14.3%) |  |
| Race, n (%) |  |  | 0.104 |
| Asian | 18 (4.3%) | 33 (8%) |  |
| Black or African American | 15 (3.6%) | 12 (2.9%) |  |
| White | 169 (40.8%) | 167 (40.3%) |  |
| Age, n (%) |  |  | 0.033 |
| <=45 | 108 (21.2%) | 133 (26.1%) |  |
| >45 | 147 (28.8%) | 122 (23.9%) |  |
| Histological type, n (%) |  |  | 0.022 |
| Classical | 178 (34.9%) | 186 (36.5%) |  |
| Follicular | 45 (8.8%) | 56 (11%) |  |
| Other | 6 (1.2%) | 3 (0.6%) |  |
| Tall Cell | 26 (5.1%) | 10 (2%) |  |
| Residual tumor, n (%) |  |  | 0.066 |
| R0 | 191 (42.6%) | 199 (44.4%) |  |
| R1 | 31 (6.9%) | 23 (5.1%) |  |
| R2 | 4 (0.9%) | 0 (0%) |  |
| Extrathyroidal extension, n (%) |  |  | < 0.001 |
| No | 151 (30.7%) | 187 (38%) |  |
| Yes | 98 (19.9%) | 56 (11.4%) |  |
| Primary neoplasm focus type, n (%) |  |  | 0.287 |
| Multifocal | 111 (22.2%) | 122 (24.4%) |  |
| Unifocal | 141 (28.2%) | 126 (25.2%) |  |
| Neoplasm location, n (%) |  |  | 0.330 |
| Bilateral | 38 (7.5%) | 50 (9.9%) |  |
| Isthmus | 11 (2.2%) | 11 (2.2%) |  |
| Left lobe | 86 (17.1%) | 91 (18.1%) |  |
| Right lobe | 118 (23.4%) | 99 (19.6%) |  |
| Thyroid gland disorder history, n (%) |  |  | 0.524 |
| Lymphocytic Thyroiditis | 32 (7.1%) | 42 (9.3%) |  |
| Nodular Hyperplasia | 34 (7.5%) | 34 (7.5%) |  |
| Normal | 141 (31.2%) | 144 (31.9%) |  |
| Other, specify | 15 (3.3%) | 10 (2.2%) |  |
| OS event, n (%) |  |  | 1.000 |
| Alive | 247 (48.4%) | 247 (48.4%) |  |
| Dead | 8 (1.6%) | 8 (1.6%) |  |
| PFI event, n (%) |  |  | 0.003 |
| Alive | 217 (42.5%) | 239 (46.9%) |  |
| Dead | 38 (7.5%) | 16 (3.1%) |  |
| Age, median (IQR) | 49 (35, 61) | 45 (35, 56) | 0.092 |

**Supplementary Table 4.** Association between OCA2 expression and clinicopathologic features in the validation cohort.
