# Supplementary material for: Key molecules associated with thyroid carcinoma prognosis: A study based on transcriptome sequencing and GEO datasets
Source: Front Immunol. 2022 Aug 17;13:964891. doi: 10.3389/fimmu.2022.964891 (PMC9428590; doi:10.3389/fimmu.2022.964891)
Supplement: Supplementary file 6 [file Table_5.docx]

| Characteristics | Total(N) | Univariate analysis | |  | Multivariate analysis | |
| --- | --- | --- | --- | --- | --- | --- |
|  |  | Hazard ratio (95% CI) | P value |  | Hazard ratio (95% CI) | P value |
| T stage | 508 |  |  |  |  |  |
| T1 | 143 | Reference |  |  |  |  |
| T2 | 167 | 3.622 (1.218-10.768) | **0.021** |  | 2.036 (0.502-8.250) | 0.319 |
| T3 | 175 | 5.345 (1.865-15.319) | **0.002** |  | 1.934 (0.373-10.026) | 0.432 |
| T4 | 23 | 9.909 (2.896-33.909) | **<0.001** |  | 0.482 (0.049-4.771) | 0.533 |
| N stage | 460 |  |  |  |  |  |
| N0 | 229 | Reference |  |  |  |  |
| N1 | 231 | 1.658 (0.936-2.934) | 0.083 |  | 0.824 (0.353-1.926) | 0.655 |
| M stage | 295 |  |  |  |  |  |
| M0 | 286 | Reference |  |  |  |  |
| M1 | 9 | 7.305 (2.780-19.197) | **<0.001** |  | 5.838 (1.163-29.316) | **0.032** |
| Pathologic stage | 508 |  |  |  |  |  |
| Stage I | 286 | Reference |  |  |  |  |
| Stage II | 52 | 1.355 (0.508-3.611) | 0.544 |  | 1.520 (0.378-6.110) | 0.555 |
| Stage III | 113 | 2.181 (1.130-4.210) | **0.020** |  | 3.199 (1.074-9.528) | **0.037** |
| Stage IV | 57 | 4.021 (1.996-8.102) | **<0.001** |  | 4.474 (1.127-17.761) | **0.033** |
| Gender | 510 |  |  |  |  |  |
| Female | 371 | Reference |  |  |  |  |
| Male | 139 | 1.694 (0.975-2.945) | 0.062 |  | 1.077 (0.472-2.460) | 0.860 |
| Extrathyroidal extension | 492 |  |  |  |  |  |
| No | 338 | Reference |  |  |  |  |
| Yes | 154 | 1.874 (1.092-3.216) | **0.023** |  | 0.961 (0.313-2.957) | 0.945 |
| Residual tumor | 448 |  |  |  |  |  |
| R0 | 390 | Reference |  |  |  |  |
| R1 | 54 | 1.618 (0.756-3.462) | 0.215 |  |  |  |
| R2 | 4 | 2.029 (0.277-14.850) | 0.486 |  |  |  |
| RGS8 | 510 |  |  |  |  |  |
| Low | 255 | Reference |  |  |  |  |
| High | 255 | 0.381 (0.210-0.691) | **0.001** |  | 0.589 (0.215-1.615) | 0.304 |
| DGKI | 510 |  |  |  |  |  |
| Low | 255 | Reference |  |  |  |  |
| High | 255 | 0.517 (0.293-0.910) | **0.022** |  | 0.918 (0.321-2.629) | 0.873 |
| OCA2 | 510 |  |  |  |  |  |
| Low | 255 | Reference |  |  |  |  |
| High | 255 | 0.406 (0.227-0.729) | **0.003** |  | 0.869 (0.367-2.058) | 0.750 |

**Supplementary Table 5.** Univariate and multivariate Cox proportional hazards analysis of RGS8, DGKI and OCA2 expression and PFI for patients with THCA in the validation cohort.
